# Supplementary figures and images for: Detecting clinically actionable variants in the 3′ exons of PMS2 via a reflex workflow based on equivalent hybrid capture of the gene and its pseudogene
Source: BMC Med Genet. 2018 Sep 29;19:176. doi: 10.1186/s12881-018-0691-9 (PMC6162901; doi:10.1186/s12881-018-0691-9)

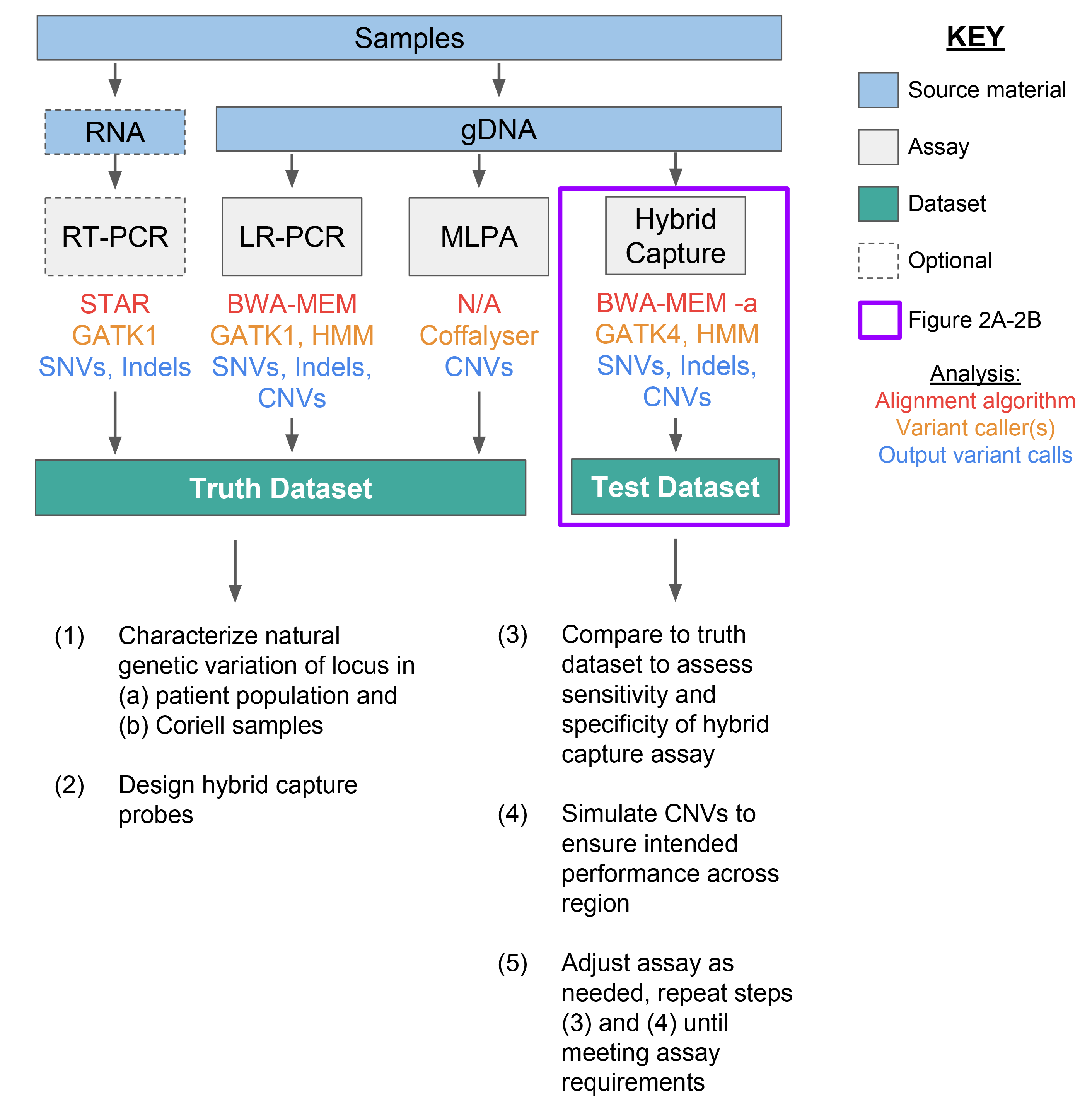

Supplement: Supplementary file 1 — Figure S1. Orthogonal datasets used to build the assay. Diagram demonstrating the assays, datasets, algorithms, and analyses used to build the hybrid capture assay for the last five exons of PMS2. The Coriell samples (1b) can be used by other researchers without repeating the LR-PCR as we have made those data publicly available (accession #PRJEB27948, see Declarations). Genomic DNA (gDNA). (PNG 219 kb) [file 12881_2018_691_MOESM1_ESM.png]

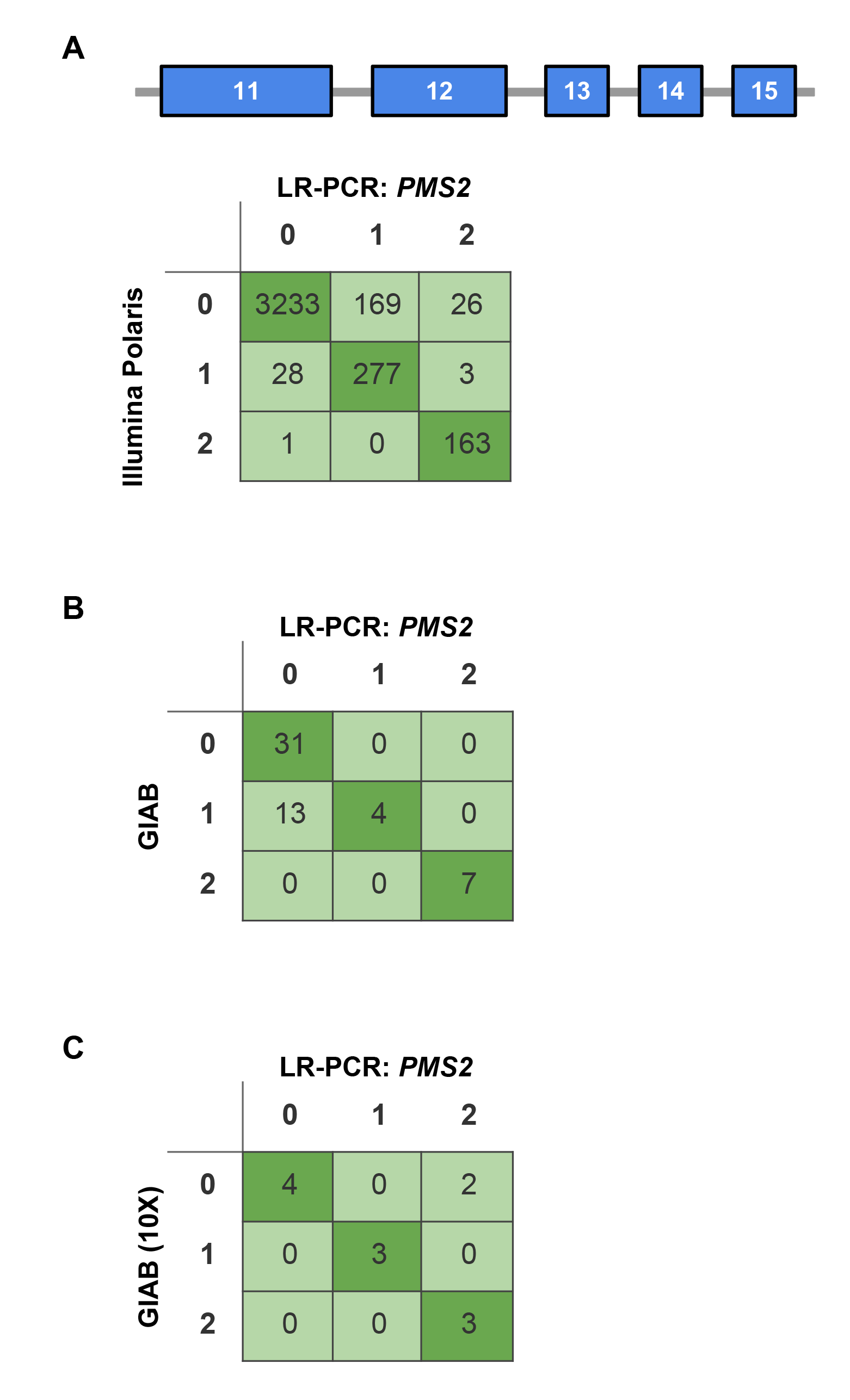

Supplement: Supplementary file 4 — Figure S2. PMS2 exons 11–15 reference genotypes (from Polaris and GIAB) are inconsistent with PMS2 LR-PCR. (A) Concordance between LR-PCR variant calls and Polaris variant calls. (B) Concordance between LR-PCR variant calls and the GIAB multisample call set (including high confidence and filtered variant calls) for all five GIAB samples. (C) Concordance between LR-PCR variant calls and the 10X Genomics haplotype call set available for four GIAB samples. (PNG 76 kb) [file 12881_2018_691_MOESM4_ESM.png]

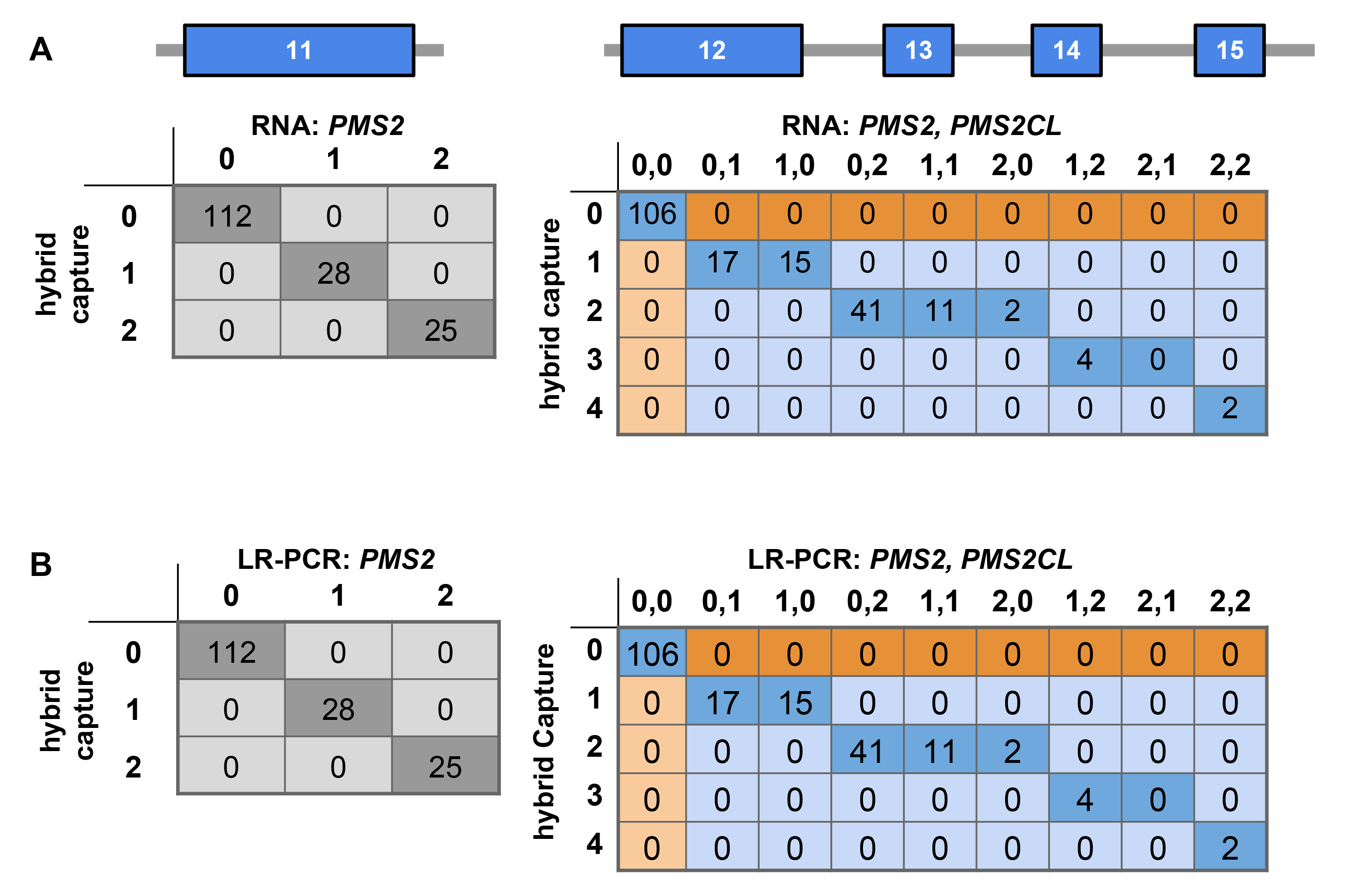

Supplement: Supplementary file 5 — Figure S3. RNA data corroborate hybrid capture and LR-PCR data. (A) Concordance between hybrid capture data and RT-PCR (RNA) for PMS2 and PMS2CL. (B) Concordance between hybrid capture data and LR-PCR (DNA) for PMS2 and PMS2CL. (PNG 135 kb) [file 12881_2018_691_MOESM5_ESM.png]
